# COVID‐19 infection and pain in adolescents with sickle cell disease: A case series

**DOI:** 10.1002/jha2.587

**Published:** 2022-10-06

**Authors:** Heidi M. Heyman, Nicole M. Alberts, Matthew Rees, Latika Puri, Michael J. Frett, Doralina L. Anghelescu

**Affiliations:** ^1^ Department of Pediatric Medicine Division of Anesthesiology St. Jude Children's Research Hospital Memphis Tennessee USA; ^2^ Department of Psychology Concordia University Montréal Quebec Canada; ^3^ Department of Oncology St. Jude Children's Research Hospital Memphis Tennessee USA; ^4^ Department of Hematology Loma Linda University Loma Linda California USA

**Keywords:** anemia, chronic pain, COVID‐19, neuropathic pain, sickle cell disease, vaso‐occlusive crisis

## Abstract

Adolescents with sickle cell disease (SCD) have been shown to have pain‐related sequelae following COVID‐19 infection. In this case series, we discuss five adolescents with SCD and SARS‐CoV‐2 infection who subsequently developed complex pain circumstances manifested as: (1) increased frequency of acute care visits or admissions for pain; (2) new onset chronic pain; (3) new onset neuropathic pain; (4) escalation in the complexity of pharmacologic therapies; (5) increased use of nonpharmacologic interventions. While more research is needed to fully understand the implications of COVID‐19 infection on pain in adolescents with SCD, these cases suggest the presence of a complex relationship.

## 1 INTRODUCTION

Sickle cell disease (SCD) places patients at risk for severe illness from COVID‐19 [1, 2]. The pathophysiology of COVID‐19 in patients with SCD leads to pulmonary effects and hypoxia‐induced erythrocyte sickling. Both conditions associate hyperinflammatory states and susceptibility to coagulopathy and thrombosis [3]. SCD often leads to an immunocompromised state, due to splenic auto‐infarction or surgical splenectomy, and secondary organ dysfunction poses risks for significant morbidity and mortality [4].

COVID‐19 has been associated with acute and chronic pain in the general population [5, 6]; in patients with SCD, acute pain is reportedly the most common presenting symptom of COVID‐19 [7]. COVID‐19 may cause peripheral and central neurological complications [8, 9, 10], including neuropathic pain (NP), not unlike other viral infections [11, 12, 13, 14]. Pain from vaso‐occlusive crisis (VOC) and acute chest syndrome (ACS) as presenting symptoms of COVID‐19 infection, and increased frequency and severity of VOC pain crises after COVID‐19 infection have been reported [2, 7, 15–19].

This case series describes changes in pain characteristics in five adolescents with SCD after COVID‐19 infection. Our aims are: (1) to describe the history, presentation, and clinical course of pain after COVID‐19 infection; (2) to present the escalation of pain treatment plans; (3) to draw attention to the relationship between COVID‐19 infection and pain in adolescents with SCD.

## 2 METHODS

A retrospective review identified five adolescents with SCD referred to the Pain Management Service at St. Jude Children's Research Hospital in Memphis, TN, for complex pain after COVID‐19 infection, between March 2020 and August 2021. Pain characteristics and escalation of treatment lines were noted:

1. Increased frequency of acute pain visits and number of inpatient‐days for pain, when comparing 12‐months pre‐ and post‐COVID‐19 infection;
2. Onset of chronic pain (exceeding 3 months);
3. Onset of NP (supported by painDETECT scores);[20]
4. Pharmacologic interventions, either NP‐directed or addressing central sensitization and opioid‐induced hyperalgesia;
5. Nonpharmacological intervention for pain.

## 3 RESULTS AND CASE PRESENTATIONS

Patient demographics, changes in pain characteristics, and therapies are outlined in Table 1. Changes in pain intensity and pain descriptors are outlined in Table 2.

**TABLE 1:** Changes in pain patterns and complex treatment plans in five adolescents with sickle cell disease (SCD) after COVID‐19 infection

**Case^#^**	**Patient demographics**	**Inpatient days for pain pre‐ vs. post‐COVID** *	**New onset chronic pain** **	**New onset neuropathic pain** ***	**Complex pharmacologic therapies** ****	**Complex nonpharmacological interventions** *****
**1**	16yo M with HbSS	6 vs. 22	Yes	Yes	Methadone, pregabalin, amitriptyline, clonidine	Diaphragmatic breathing, guided imagery, grounding techniques, CBT
**2**	17yo F with HbSS	0 vs. 57	Yes	Yes	Meloxicam, long‐acting morphine, oxycodone, lidocaine infusions, ketamine infusions, methadone, gabapentin, amitriptyline, duloxetine	Guided‐imagery, diaphragmatic breathing, nutrition counseling, massage therapy, acupressure, acupuncture, VR
**3**	17yo F with HbSS	10 vs. 78	Yes	Yes	Meloxicam, oxycodone, cyclobenzaprine, methadone, gabapentin, lidocaine patch, buprenorphine patch	Diaphragmatic breathing, guided imagery, CBT, yoga, heat and physical therapy
**4**	17yo M with HbSS	12 vs. 11	Yes	Yes	Pregabalin, duloxetine, opioids, acetaminophen, NSAIDs, ketamine infusion, buprenorphine patch, oxycodone, methadone	Psychoeducation, CBT, VR, meditation, heat and massage therapy, acupuncture, auricular acupressure therapy
**5**	14yo F with HbSC	0 vs. 21	Yes	None reported	Acetaminophen, ibuprofen, long‐acting morphine, oxycodone, lidocaine patch, buprenorphine patch	Diaphragmatic breathing, CBT, massage, acupuncture, yoga

* Days of admission for pain, comparing 12‐month preceding and following the COVID‐19 infection (case #4 was lost to follow‐up 4 months of post‐COVID due to transfer to adult care).

** Pain lasting longer than 3 months.

*** When available, painDETECT scores were included in the assessment of neuropathic pain. The painDETECT questionnaire is a validated, symptom‐based assessment tool developed to assist in the diagnosis of neuropathic pain: likely neuropathic pain (score **≥**19), unclear (score 13–18), or likely non‐neuropathic (nociceptive) pain (score **≤**12).

**** Therapy addressed either neuropathic pain (gabapentinoids, tricyclic antidepressants, methadone), or central sensitization and opioid‐induced hyperalgesia (methadone, ketamine infusions, lidocaine infusions, buprenorphine).

***** Including consults from psychology, psychiatry, integrative medicine, and child life specialists.

**TABLE 2:** Changes in pain intensity and patient‐reported pain descriptors in five adolescents with sickle cell disease (SCD) after COVID‐19 Infection

**Case ^#^**	**Patient demographics**	**Pain intensity (NPRS** * **) pre‐COVID** **	**Pain intensity (NPRS** * **) post‐COVID** **	**Patient‐reported pain descriptions post‐COVID** ***	**Neuropathic pain (painDETECT scores** **** **) pre‐COVID and timing**	**Neuropathic pain (painDETECT scores** **** **) post‐COVID and timing**
**1**	16yo M with HbSS	8–10	0–10	“knife‐stabbing”, “frozen” “numbness”, “burning” “throbbing”	Not reported	7 (1 month)
**2**	17yo F with HbSS	7–10	0–10	“achy”, “sharp”, “throbbing”	Not reported	28 (2 months) 24 (13 months)
**3**	17yo F with HbSS	7–9.5	6–12	“stabbing”, “electrical”, “sharp”, “shooting”, “like my bones are breaking”	Not reported	8 (3 months) 22 (4 months)
**4**	17yo M with HbSS	5–9	0–10	“deep”, “achy”, “sharp”	2 (18 months) 0 (7 months)	22 (2 weeks)
**5**	14yo F with HbSC	None reported	0–8.5	“aching”, “cramping”, “sore”	Not reported	5 (2 months)

* NPRS‐Numerical Pain Rating Scale ranging from 0 (no pain at all) to 10 (worst imaginable pain).

** Pain scores reported during acute care visits or admissions in the 12‐month preceding versus following COVID‐19 infection.

*** Descriptors gathered from pain service notes following COVID‐19 infection.

**** As documented on a scale of 0 to 35, with scores **≥**19 suggestive of neuropathic pain, scores **≤**12 suggestive of non‐neuropathic (nociceptive pain), and scores 13–18 suggestive of unclear neuropathic versus non‐neuropathic pain.

### 3.1 Patient 1

This 16‐year‐old male with SCD (HbSS) was found to have SARS‐CoV‐2 antibodies following a right hip core decompression for SCD‐related avascular necrosis. Postoperative complications included lower extremity deep venous thrombosis, pulmonary emboli, ACS, VOC, and need for ventilatory support. He subsequently developed feet pain described as “knife stabbing,” “frozen,” “numbness,” “burning,” and “throbbing,” suggestive of NP, despite the absence of a high painDETECT score. Pain medications included methadone, pregabalin, amitriptyline, and clonidine. Nonpharmacological therapies for pain included diaphragmatic breathing, guided imagery, grounding techniques, and cognitive behavioral therapy (CBT). A year later, he had been weaned off from analgesic medications.

### 3.2 Patient 2

This 17‐year‐old female with SCD (HbSS) went into premature labor at 36‐weeks after developing symptoms of cough, fever, nausea, and vomiting, with a positive COVID‐19 polymerase chain reaction (PCR) test. Subsequently, she developed severe, generalized pain, described as “achy,” “sharp,” and “throbbing,” with a painDETECT score suggestive of NP. Pain was refractory to meloxicam and opioids at home, leading to admission and treatment with gabapentin, long‐acting morphine, oxycodone, and lidocaine infusions. For persistent pain following discharge, long‐acting morphine was rotated to methadone, and buprenorphine patch and cyclobenzaprine were added. She reported improvement, facilitating opioid weaning. During the subsequent 11 months, she returned for acute care visits for pain and nine admissions for VOC pain, for 3 to 11 days each admission. Medications for pain included lidocaine and ketamine infusions, methadone, gabapentin, amitriptyline, and duloxetine. Nonpharmacological interventions included guided‐imagery, diaphragmatic breathing, diet and nutrition counseling, massage therapy, acupressure, acupuncture, and virtual reality (VR) sessions. A year later, she continued follow‐up with the pain service for VOC pain episodes, recurrent admissions for pain, and NP symptoms, as suggested by a painDETECT score of 24, while treated with methadone, gabapentin, duloxetine, acetaminophen, meloxicam, and morphine.

### 3.3 Patient 3

This 17‐year‐old female with SCD (HbSS) presented with fever, malaise, sore throat, and VOC pain, and a positive COVID‐19 PCR test. Subsequently, she reported worsening pain in the lower extremities, back, and chest. Six weeks later, she presented with similar pain crisis and another positive COVID‐19 PCR test, despite two negative COVID‐19 tests between these two pain crises. A few weeks later, she presented with a third VOC crisis, describing pain as “deep,” “stabbing,” “like my bones are breaking.” Two months later, she continued to report pain with “electrical,” “sharp,” “shooting” characteristics, and a painDETECT score of 22, consistent with NP. Pain management included meloxicam, oxycodone, cyclobenzaprine, methadone, gabapentin, lidocaine, and buprenorphine patches. Nonpharmacologic therapies included diaphragmatic breathing, guided imagery, CBT, yoga, heat, and physical therapy. Subsequently, she had five acute care visits and two pain‐related hospitalizations before medical care was transitioned to adult services.

### 3.4 Patient 4

This 17‐year‐old male with SCD (HbSS) presented with increased severity of pre‐existing chronic back pain in conjunction with COVID‐19 infection, confirmed by SARS‐CoV‐2 antibodies. Over the following 3 months, the back pain progressed to a “deep,” “sharp,” and “achy” generalized body pain, with frequent exacerbations and acute care visits. A painDETECT score of 22 suggested NP, and pregabalin and duloxetine were started. During admissions, pain was treated with opioids, acetaminophen, NSAIDs, and low‐dose ketamine infusions. Upon discharge, he was prescribed buprenorphine patch and oxycodone; followed by a rotation to methadone, with symptom improvement. Nonpharmacologic therapies included psychoeducation, CBT, VR sessions, meditation, heat and massage therapy, acupuncture, and auricular acupressure therapy. A year later, he remained on methadone, pregabalin, duloxetine, oxycodone, acetaminophen, and NSAIDs, with pain scores higher than his pre‐COVID baseline.

### 3.5 Patient 5

This 14‐year‐old female with HbSC was hospitalized with pneumonia and ACS, requiring supplemental oxygen and exchange transfusion, in conjunction with a positive COVID‐19 PCR test. Two weeks after discharge, she presented with worsening back and chest pain and was re‐admitted for pain, fever, and ACS. In the subsequent 5 months, she presented for five acute care visits or admissions and continued to report back pain, described as “aching,” “cramping,” and “sore” and “different and worse” compared to her pre‐COVID joint pain. Despite absence of a painDETECT score suggestive of NP, pain was refractory to standard pain regimens (acetaminophen, ibuprofen, long‐acting morphine). Additional medications included oxycodone, lidocaine, and buprenorphine patches. Nonpharmacologic therapies included diaphragmatic breathing, CBT, massage, acupuncture, and yoga. Six months later, she continued to report back pain, treated with buprenorphine patches and oxycodone.

## 4 DISCUSSION

These five cases suggest that COVID‐19 infection may exacerbate pain in adolescents with SCD. The association of pain and COVID‐19 may be supported by the relationship between neurological and musculoskeletal manifestations and the expression and distribution of angiotensin‐converting enzyme 2, which was identified as a functional receptor for COVID‐19 [3, 21]. The inflammatory cytokine interleukin 6 plays a critical role in the pathogenesis of NP and inflammatory pain [22] and has been associated with poor clinical outcomes in SCD and with more severe COVID‐19 infections [23]. While definite clinical and pathological evidence for neurotropism of the SARS‐COV‐2 virus is lacking [8], a correlation between COVID‐19 infection and new onset or exacerbation of small fiber neuropathy has been suggested [24, 25]. These associations suggest a complex interplay between the pathophysiology of these two diseases and the pain experience.

### 4.1 Frequency of acute care visits

Four of the five patients in this case series had increased frequency in acute care visits following COVID‐19 infection. High healthcare utilization has financial, economic, and systemic impacts. SCD is marked by high utilization of medical resources [26], defined as more or equal to three events per 12 months [27]. Our series indicate an increased utilization of healthcare resources following COVID‐19 infection. The transition to adult care is challenging, with increased frequency of healthcare visits and higher mortality rates, highlighting the importance of mitigating increases in acute care visits in adolescents [28, 29, 30, 31].

### 4.2 Chronic pain

All five patients were treated for chronic pain following COVID‐19 infection. Proposed mechanisms may include chronic pain as a postviral syndrome, as a result of viral‐associated organ damage, as exacerbation of pre‐existing pain, or as a result of exacerbation of psychosocial risk factors such as inactivity, fear, anxiety, and depression [5, 32, 33]. The diagnosis of chronic pain, irrespective to SCD, is associated with high rates of health care utilization [34]. Patients with chronic pain are more likely to need opioid therapy, with associated risks of misuse, addiction, and overdose [35, 36, 37].

### 4.3 NP

While NP is rarely reported with COVID‐19 [9], our cases suggest that NP may be a sequela of COVID‐19 infection in patients with SCD. Defined by the International Association for the Study of Pain as “pain caused by a lesion or disease of the somatosensory nervous system” [38], NP is characterized by allodynia, hyperalgesia, and sudden pain attacks, as outlined by the validated painDETECT screening questionnaire [20]. NP may be chronic, debilitating, may be unresponsive to traditional pain therapies, and often presents with concurrent psychiatric comorbidities, such as depression [33, 39].

A recent study evaluating NP in youth with SCD indicated an association between higher healthcare utilization and prevalence of NP [40]. Frequent acute pain episodes may contribute to central sensitization and hyperalgesia and create a basis for NP mechanisms. Early diagnosis of NP is important, considering that NP has substantial impact on health‐related quality of life and necessitates specific lines of therapy [41].

### 4.4 Pharmacologic therapies for pain

Standard therapies for SCD‐related pain include nonsteroidals, acetaminophen, and opioids. Our series suggest that pain in patients with SCD after COVID‐19 infection seems “opioid‐resistant” and may require complex pharmacologic regimens, including: NP‐targeted medications (gabapentinoids and tricyclic antidepressants), medications aimed to reduce opioid exposure, central sensitization, and opioid‐induced hyperalgesia (lidocaine and ketamine infusions), and medications effective for both purposes (methadone and buprenorphine). Interventions to address central sensitization and hyperalgesia may reduce opioid use and associated side effects.

### 4.5 Nonpharmacologic therapies for pain

Nonpharmacological pain management involved multidisciplinary teams, including psychology, psychiatry, child life specialists, integrative medicine, physical therapy, and nutritionists. Therapies utilized included psychoeducation, CBT, guided imagery, grounding techniques, diaphragmatic breathing, meditation, VR, nutrition counseling, physical therapy, yoga, massage therapy, heat therapy, acupuncture, and acupressure. Considering the complex nature of pain and the exacerbation of psychosocial stressors associated with the COVID‐19 pandemic, the emphasis on biopsychosocial approaches is beneficial.

## 5 LIMITATIONS

This case series is limited to a small sample, limited patient age (14–17 years old), and incomplete long‐term follow‐up. While the temporal relationship between COVID‐19 infection and changes in pain characteristics is suggestive of their relationship, we acknowledge that pain from SCD evolves regardless of COVID‐19 infection. We did not examine socioeconomic factors that may have led to differences in clinical outcomes, nor did we address the role of caregivers and other family factors in exacerbating, relieving, or maintaining pain.

## 6 CONCLUSION

These cases suggest the association of COVID‐19 infection in adolescents with SCD with severe pain outcomes, increased frequency of acute care visits and/or hospitalization for pain, new onset chronic pain or NP, and escalation in complexity of pharmacologic and nonpharmacologic interventions. The complex relationships between SCD, COVID‐19 infection, and pain suggest the importance of future research in their association.

## CONFLICT OF INTEREST

The authors have no conflict of interest to report.

## ETHICS STATEMENT

This manuscript, including any related work from the same study, has not been submitted elsewhere or been previously published. All authors have significantly contributed to the manuscript, have reviewed, and agreed upon the manuscript content, and there is no conflict of interest to disclose. This study has received IRB approval under the Sickle Cell Clinical Research and Intervention Program (SCCRIP) at St. Jude Children's Research Hospital. Informed consent was obtained as indicated via the IRB approval.

## Data Availability

The data that support the findings of this study are available from the corresponding author, DA, upon reasonable request.
